# Supplementary material for: Genetic characterization for lesion mimic and other traits in relation to spot blotch resistance in spring wheat
Source: PLoS One. 2020 Oct 5;15(10):e0240029. doi: 10.1371/journal.pone.0240029 (PMC7535040; doi:10.1371/journal.pone.0240029)
Supplement: S1 Table — (DOCX) [file pone.0240029.s001.docx]

**Supplementary Table 1: Marker distribution among the population studied**

| **Chr.** | **Size (Mb)** | **No. of SNP** | **Average number of SNP per Mb** | **Chr. LD** |
| --- | --- | --- | --- | --- |
|  |  |  |  |  |
| **1A** | 594.1 | 604 | 1 | 0.28 |
| **1B** | 689.85 | 1304 | 2 | 0.42 |
| **1D** | 495.45 | 407 | 1 | 0.445 |
| **2A** | 780.8 | 801 | 1 | 0.426 |
| **2B** | 801.26 | 1469 | 2 | 0.404 |
| **2D** | 651.85 | 431 | 1 | 0.415 |
| **3A** | 750.84 | 625 | 1 | 0.402 |
| **3B** | 830.83 | 915 | 1 | 0.393 |
| **3D** | 615.55 | 119 | 1 | 0.389 |
| **4A** | 744.59 | 550 | 1 | 0.384 |
| **4B** | 673.62 | 390 | 1 | 0.375 |
| **4D** | 509.86 | 45 | 1 | 0.35 |
| **5A** | 709.77 | 835 | 1 | 0.368 |
| **5B** | 713.15 | 1296 | 1 | 0.365 |
| **5D** | 566.08 | 141 | 1 | 0.362 |
| **6A** | 618.08 | 808 | 1 | 0.33 |
| **6B** | 720.99 | 1047 | 2 | 0.332 |
| **6D** | 473.59 | 143 | 1 | 0.33 |
| **7A** | 736.71 | 744 | 1 | 0.313 |
| **7B** | 750.62 | 815 | 1 | 0.319 |
| **7D** | 638.69 | 100 | 1 | 0.317 |
