# Supplementary material for: Genetic characterization for lesion mimic and other traits in relation to spot blotch resistance in spring wheat
Source: PLoS One. 2020 Oct 5;15(10):e0240029. doi: 10.1371/journal.pone.0240029 (PMC7535040; doi:10.1371/journal.pone.0240029)
Supplement: S2 Table — (DOCX) [file pone.0240029.s002.docx]

**Supplementary Table 2: Distribution of 13589 highly polymorphic SNPS throughout the wheat genome**

| **Chromosomes** | **Wheat genome** | |  | **Total** |
| --- | --- | --- | --- | --- |
|  | **A** | **B** | **D** |  |
| **1** | 604 | 1304 | 407 | 2315 |
| **2** | 801 | 1469 | 431 | 2701 |
| **3** | 625 | 915 | 119 | 1659 |
| **4** | 550 | 390 | 45 | 985 |
| **5** | 835 | 1296 | 141 | 2272 |
| **6** | 808 | 1047 | 143 | 1998 |
| **7** | 744 | 815 | 100 | 1659 |
| **Total** | 4967 | 7236 | 1386 | 13589 |
