# Supplementary material for: Genetic characterization for lesion mimic and other traits in relation to spot blotch resistance in spring wheat
Source: PLoS One. 2020 Oct 5;15(10):e0240029. doi: 10.1371/journal.pone.0240029 (PMC7535040; doi:10.1371/journal.pone.0240029)
Supplement: S3 Table — (DOCX) [file pone.0240029.s003.docx]

**Supplementary Table 3: List of lines in the spring wheat association mapping (WAMI) evaluated over three years in BHU, Varanasi.**

| **S.No.** | **Entry** | **Pedigree** |
| --- | --- | --- |
| 1 | 9001 | COOK/VEE//DOVE/SERI/3/BJY/COC |
| 2 | 9002 | JUP/ZP//COC/3/PVN/4/GEN |
| 3 | 9003 | PFAU/VEE#5 |
| 4 | 9004 | SERI M 82 |
| 5 | 9005 | VORONA/GEN |
| 6 | 9006 | KAUZ/GEN |
| 7 | 9007 | KEA/TOW//LIRA |
| 8 | 9008 | LIRA/URES//MILO 9G19-2-26 |
| 9 | 9009 | MYNA/VUL//PRL |
| 10 | 9011 | TIA.3 |
| 11 | 9012 | CHOIX M 95 |
| 12 | 9013 | KAUZ*2/FN//KAUZ |
| 13 | 9014 | KAUZ*2/MNV//KAUZ |
| 14 | 9015 | PAT10/ALD//PAT72300/3/PVN/4/URES/5/PFAU |
| 15 | 9016 | PRINIA |
| 16 | 9017 | CAR422/ANA//URES |
| 17 | 9018 | CIANO T 79 |
| 18 | 9019 | HD2206/HORK//BUC/BUL |
| 19 | 9020 | PAT10/ALD//PAT72300/3/PVN/4/BOW |
| 20 | 9021 | VORONA/CNO79 |
| 21 | 9022 | AZ//KAL/BB/3/PGO |
| 22 | 9023 | BACANORA T 88 |
| 23 | 9024 | FCT/3/GOV/AZ//MUS/4/DOVE/BUC |
| 24 | 9025 | INIFAP M 97 |
| 25 | 9026 | KAUZ*2//DOVE/BUC/3/KAUZ |
| 26 | 9027 | KAUZ*2/TRAP//KAUZ |
| 27 | 9028 | LIRA/BUC |
| 28 | 9029 | PARA2//JUP/BJY/3/VEE/JUN/4/2*KAUZ |
| 29 | 9030 | RHEA |
| 30 | 9031 | TOBARITO M 97 |
| 31 | 9032 | TRAP#1/BOW |
| 32 | 9033 | TURACO/CHIL |
| 33 | 9034 | KAUZ*3//TC*6/RL5406(RL6043) |
| 34 | 9035 | KAUZ*2//TC*6/RL6081/3/KAUZ |
| 35 | 9036 | KAUZ*2/YACO//KAUZ |
| 36 | 9037 | PRL/VEE#6 |
| 37 | 9038 | UP 2338 |
| 38 | 9039 | ATTILA//ALTAR 84/AOS/3/ATTILA |
| 39 | 9040 | FANG60/7C |
| 40 | 9041 | HP 1761 |
| 41 | 9042 | HYBRID DELHI 2172 |
| 42 | 9043 | KAUZ//ALTAR 84/AOS |
| 43 | 9044 | MNCH/3*BCN |
| 44 | 9045 | RAYON F 89 |
| 45 | 9046 | SERI/7C |
| 46 | 9047 | SERI/NKT//2*KAUZ |
| 47 | 9048 | STAR//KAUZ/STAR |
| 48 | 9049 | TILHI |
| 49 | 9051 | URES/RAYON |
| 50 | 9052 | COMARA/TEG//WEAVER/3/LAJ3302 |
| 51 | 9053 | PICUS/4/CS(5A)/5RL-1//BUC/BJY/3/ALD/PVN/5/LAJ3302 |
| 52 | 9054 | TIA.2/KAUZ |
| 53 | 9055 | HP 1731 |
| 54 | 9056 | IAS62/ALDAN//2*SKAUZ |
| 55 | 9057 | KAUZ/RAYON |
| 56 | 9058 | KEA/TAN/4/TSH/3/KAL/BB//TQFN/5/PAVON/6/SW89.3064 |
| 57 | 9059 | PASTOR/2*SITTA |
| 58 | 9060 | BHRIKUTI |
| 59 | 9061 | BL 1724 |
| 60 | 9062 | BOW/PRL*3/6/WRM/4/FN/3*TH//K58/2*N/3/AUS-6869/5/PELOTAS-ARTHUR/7/HE1/3*CNO79//2*SERI |
| 61 | 9063 | BUC/PRL//WEAVER |
| 62 | 9064 | CHUM18/5*BCN |
| 63 | 9065 | LAJ3302/2*MO88 |
| 64 | 9066 | MILAN/3/JUP/BJY//URES |
| 65 | 9067 | NL 750 |
| 66 | 9068 | PUNJAB 96 |
| 67 | 9069 | RABE/2*MO88 |
| 68 | 9070 | SW89-5124*2/FASAN |
| 69 | 9071 | TIA.4/WL6572//RL6043/3*GEN/3/LUAN |
| 70 | 9072 | CAZO/KAUZ//KAUZ |
| 71 | 9073 | CHIR1//SHA5/WEAVER |
| 72 | 9074 | HUITES/4/CS/TH.SC//3*PVN/3/MIRLO/BUC |
| 73 | 9075 | KAUZ//BOW/NKT |
| 74 | 9076 | KAUZ/WEAVER |
| 75 | 9077 | MINO |
| 76 | 9078 | OASIS/SKAUZ//4*BCN |
| 77 | 9079 | SHA3/SERI//SHA4/LIRA/3/CHIR1/4/SHA7//PRL/VEE#6/3/FASAN |
| 78 | 9080 | SW89.5181/KAUZ |
| 79 | 9081 | W462//VEE/KOEL/3/PEG//MRL/BUC |
| 80 | 9082 | GUAM92/KAUZ |
| 81 | 9083 | CHEN/AEGILOPS SQUARROSA (TAUS)//BCN/3/2*KAUZ |
| 82 | 9084 | CHEN/AEGILOPS SQUARROSA (TAUS)//BCN/3/KAUZ |
| 83 | 9085 | CMH84.3379/CMH78.578//MILAN |
| 84 | 9086 | HUAYTU CIAT |
| 85 | 9087 | OTUS |
| 86 | 9088 | TARACHI F 2000 |
| 87 | 9089 | SITE/MO/4/NAC/TH.AC//3*PVN/3/MIRLO/BUC |
| 88 | 9090 | SKAUZ*2/FCT |
| 89 | 9091 | SURUTU-CIAT |
| 90 | 9092 | TAURUM |
| 91 | 9093 | INQALAB 91 |
| 92 | 9094 | SKAUZ*2/FCT |
| 93 | 9095 | CNDO/R143//ENTE/MEXI_2/3/AEGILOPS SQUARROSA (TAUS)/4/WEAVER/5/2*KAUZ |
| 94 | 9096 | KETUPA*2/PASTOR |
| 95 | 9097 | WEAVER/3/SAPI/TEAL//HUI/4/CROC_1/AE.SQUARROSA (213)//PGO/5/SKAUZ*2/SRMA |
| 96 | 9098 | KAUZ*2/TRAP//KAUZ/3/PASTOR/4/SKAUZ*2/SRMA |
| 97 | 9099 | REH/HARE//2*BCN/3/CROC_1/AE.SQUARROSA (213)//PGO/4/HUITES |
| 98 | 9101 | ATTILA/3*BCN//BAV92/3/TILHI |
| 99 | 9102 | CROC_1/AE.SQUARROSA (205)//BORL95/3/PASTOR |
| 100 | 9103 | CHEN/AE.SQ//WEAVER/3/SSERI1 |
| 101 | 9104 | BAV92/3/OASIS/SKAUZ//4*BCN/4/PASTOR |
| 102 | 9105 | CROC_1/AE.SQUARROSA (205)//KAUZ/3/2*KAUZ*2/YACO//KAUZ |
| 103 | 9106 | WBLL1*2/KUKUNA |
| 104 | 9107 | ROELFS F2007 |
| 105 | 9108 | HD2687 |
| 106 | 9109 | PBW450 |
| 107 | 9110 | MILAN/S87230//BAV92 |
| 108 | 9111 | ATTILA/3*BCN*2//BAV92 |
| 109 | 9112 | TOBA97/PASTOR |
| 110 | 9113 | FRET2*2/4/SNI/TRAP#1/3/KAUZ*2/TRAP//KAUZ |
| 111 | 9114 | ALTAR 84/AE.SQUARROSA (221)//3*BORL95/3/URES/JUN//KAUZ/4/WBLL1 |
| 112 | 9115 | TUKURU//BAV92/RAYON |
| 113 | 9116 | SUNSU/CHIBIA |
| 114 | 9117 | WBLL1*2/4/YACO/PBW65/3/KAUZ*2/TRAP//KAUZ |
| 115 | 9118 | WBLL1*2/VIVITSI |
| 116 | 9119 | F6.74/BUN//SIS/3/YR/PAM |
| 117 | 9120 | PASTOR |
| 118 | 9121 | GALVEZ S 87 |
| 119 | 9122 | PAVON |
| 120 | 9123 | VEE#8/5/VEE/4/KLTO//S12/J9281.67/3/MO/JUP |
| 121 | 9124 | IRENA |
| 122 | 9125 | BB//TOB/CNO67/3/HUAC/4/TI-R/3/BB/PL//SX |
| 123 | 9126 | CULIACAN T 89 |
| 124 | 9127 | KITE/PGO |
| 125 | 9128 | PSN/BOW//SERI |
| 126 | 9129 | GIM/LIRA |
| 127 | 9130 | URES/JUN//KAUZ |
| 128 | 9131 | PROINTA FEDERAL |
| 129 | 9132 | HI.1077 |
| 130 | 9133 | SITTA |
| 131 | 9134 | URES//BUC/FLK/3/KAUZ |
| 132 | 9135 | ARIVECHI M 92 |
| 133 | 9136 | TIA.1 |
| 134 | 9137 | RL6043/4*NAC |
| 135 | 9138 | F60314.76/MRL//CNO79 |
| 136 | 9139 | ATTILA |
| 137 | 9140 | PFAU/VEE#9 |
| 138 | 9141 | BAU/OPATA |
| 139 | 9142 | PRINIA |
| 140 | 9143 | GRANERO INTA |
| 141 | 9144 | HUITES F 95 |
| 142 | 9145 | KEA/BUC//FCT |
| 143 | 9146 | KITE/GLEN |
| 144 | 9147 | CHIL/BUC |
| 145 | 9148 | FILIN |
| 146 | 9149 | PRL/SARA//TSI/VEE#5 |
| 147 | 9151 | PJN/BOW//OPATA |
| 148 | 9152 | VEE/PJN//TUI |
| 149 | 9153 | PARA2//JUP/BJY/3/VEERY#5.4/JUN/4/TUI |
| 150 | 9154 | VEE/PJN//2*TUI |
| 151 | 9155 | URES/BBL//KAUZ/3/KAUZ |
| 152 | 9156 | ALTAR 84/AEGILOPS SQUARROSA (TAUS)//OPATA |
| 153 | 9157 | DUCULA//HUI/TUB/3/CAZO |
| 154 | 9158 | SHUHA |
| 155 | 9159 | PROINTA GRANAR |
| 156 | 9160 | TZPP/SERI//BUC |
| 157 | 9161 | PIFED/DERN |
| 158 | 9162 | HIDHAB |
| 159 | 9163 | W3918A/JUP |
| 160 | 9164 | JUN/GEN |
| 161 | 9165 | K134(60)/VEE//BOW/PVN |
| 162 | 9166 | ESDA/4/BD120/3/GTA/MXP//RUFF/FGO |
| 163 | 9167 | VEE#8//JUP/BJY/3/F3.71/TRM/4/BCN/5/KAUZ |
| 164 | 9168 | CNDO/R143//ENTE/MEXI_2/3/AEGILOPS SQUARROSA (TAUS)/4/WEAVER |
| 165 | 9169 | HXL-F86/2*BAU |
| 166 | 9170 | VI/PIFED//VEE#8 |
| 167 | 9171 | GOV/AZ//MUS/3/SARA |
| 168 | 9172 | MON/IMU//ALD/PVN |
| 169 | 9173 | TUI*2/MILAN |
| 170 | 9174 | KAUZ*2/BOW//KAUZ |
| 171 | 9175 | SIMORGH |
| 172 | 9176 | CAR853/COC//VEE/3/BOW/4/TUI/5/TUI |
| 173 | 9177 | NANJING 8646/KAUZ//BCN |
| 174 | 9178 | TUI/3/TMP64/TWN//SDY/4/RAYON |
| 175 | 9179 | VEE#5/SARA//DUCULA |
| 176 | 9180 | CLC89//ESDA/KAUZ/3/BJY/COC//PRL/BOW |
| 177 | 9181 | NS-732/HER//KAUZ |
| 178 | 9182 | 3VASKAR/G303.1M.1.3.2.2.2//KAUZ/3/SKAUZ/4/KAUZ |
| 179 | 9183 | TODY/3/JUP/BJY//SARA/4/TRAP#1/BOW/5/NL456/VEE#5 |
| 180 | 9184 | CROC_1/AE.SQUARROSA (205)//JUP/BJY/3/SKAUZ/4/KAUZ |
| 181 | 9185 | PASTOR/3/VEE#5//DOVE/BUC |
| 182 | 9186 | VEE#5//PF70354/MUS/3/PIFED/4/OR791432/VEE#3.2 |
| 183 | 9187 | KAUZ/5/PAT10/ALD//PAT72300/3/PVN/4/BOW |
| 184 | 9188 | MRL/BUC//LIRA/5/BB//TOB/CNO67/3/HUAC/4/TI-R/3/BB/PL//SX |
| 185 | 9189 | SAAR |
| 186 | 9190 | MNCH/3*BCN |
| 187 | 9191 | JUP/BJY//URES/3/HD2206/HORK//BUC/BUL |
| 188 | 9192 | SITE/PIOS |
| 189 | 9193 | PASTOR//SITE/MO/3/CHEN/AEGILOPS SQUARROSA (TAUS)//BCN |
| 190 | 9194 | FILIN/IRENA/5/CNDO/R143//ENTE/MEXI_2/3/AEGILOPS SQUARROSA (TAUS)/4/WEAVER |
| 191 | 9195 | CROC_1/AE.SQUARROSA (205)//KAUZ/3/ENEIDA |
| 192 | 9196 | F60314.76/MRL//CNO79/3/CHIL/PRL |
| 193 | 9197 | PASTOR/BAV92 |
| 194 | 9198 | BARBET1 |
| 195 | 9199 | MILVUS2 |
| 196 | 9201 | WEEBILL1 |
| 197 | 9202 | KAMBARA1 |
| 198 | 9203 | BABAX.1B.1B*3/PRL |
| 199 | 9204 | PEWIT1 |
| 200 | 9205 | FRET2 |
| 201 | 9206 | WEEBILL4 |
| 202 | 9207 | ATTILA*2/9/KT/BAGE//FN/U/3/BZA/4/TRM/5/ALDAN/6/SERI/7/VEE#10/8/OPATA |
| 203 | 9208 | ATTILA*2/PBW65 |
| 204 | 9209 | BABAX/KS93U76//BABAX |
| 205 | 9210 | SUJATA/SERI |
| 206 | 9211 | PASTOR/3/MUNIA//CHEN/ALTAR 84/5/CNDO/R143//ENTE/MEXI_2/3/AEGILOPS SQUARROSA (TAUS)/4/WEAVER |
| 207 | 9212 | URES/PRL//BAV92 |
| 208 | 9213 | VOROBEY |
| 209 | 9214 | SOROCA |
| 210 | 9215 | FILIN/2*PASTOR |
| 211 | 9216 | URES/JUN//KAUZ/3/BAV92 |
| 212 | 9217 | NAI60/HN7//BUC/3/PSN/BOW//TUI |
| 213 | 9218 | ATTILA*2/4/CAR//KAL/BB/3/NAC |
| 214 | 9219 | KAMBARA2 |
| 215 | 9220 | PRL/2*PASTOR |
| 216 | 9221 | PBW65/2*PASTOR |
| 217 | 9222 | ATTILA*2/PASTOR |
| 218 | 9223 | SERI*3//RL6010/4*YR/3/PASTOR/4/BAV92 |
| 219 | 9224 | PASTOR//HXL7573/2*BAU |
| 220 | 9225 | SOKOLL |
| 221 | 9226 | CROC_1/AE.SQUARROSA (213)//PGO/3/BAV92 |
| 222 | 9227 | MILAN/KAUZ//PRINIA/3/BAV92 |
| 223 | 9228 | ALTAR 84/AE.SQUARROSA (221)//PASTOR/3/PASTOR |
| 224 | 9229 | MILAN/KAUZ//PASTOR |
| 225 | 9230 | FLORKWA-1/DHARWAR DRY |
| 226 | 9231 | CNDO/R143//ENTE/MEXI_2/3/AEGILOPS SQUARROSA (TAUS)/4/WEAVER/5/PASTOR |
| 227 | 9232 | VEBOW/IRENA |
| 228 | 9233 | PASTOR/DHARWAR DRY |
| 229 | 9234 | BJY/COC//PRL/BOW/3/FRTL |
| 230 | 9235 | PASTOR//HXL7573/2*BAU |
| 231 | 9236 | SRMA/TUI//PASTOR |
| 232 | 9237 | SKAUZ/PASTOR/3/CROC_1/AE.SQUARROSA (224)//OPATA |
| 233 | 9238 | CNO79//PF70354/MUS/3/PASTOR/4/BAV92 |
| 234 | 9239 | MILAN/KAUZ/3/URES/JUN//KAUZ/4/CROC_1/AE.SQUARROSA (224)//OPATA |
| 235 | 9240 | KABY/BAV92/3/CROC_1/AE.SQUARROSA (224)//OPATA |
| 236 | 9241 | BOW//BUC/BUL/3/KAUZ/4/BAV92/5/MILAN/KAUZ |
| 237 | 9242 | PASTOR//MILAN/KAUZ/3/VEE/PJN//2*TUI |
| 238 | 9243 | BJY/COC//PRL/BOW/3/MILAN/KAUZ/4/BAV92 |
| 239 | 9244 | KAUZ/BAV92/3/BJY/COC//PRL/BOW |
| 240 | 9245 | FRAME/BUCHIN |
| 241 | 9246 | TEMPORALERA M 87*2/KONK |
| 242 | 9247 | FRAME*2/3/URES/JUN//KAUZ |
| 243 | 9248 | CROC_1/AE.SQUARROSA (224)//OPATA/3/PASTOR/4/PASTOR*2/OPATA |
| 244 | 9249 | RL6043/4*NAC//2*PASTOR |
| 245 | 9250 | JUPARE C 2001 |
| 246 | 9251 | CROC_1/AE.SQUARROSA (224)//OPATA/3/PASTOR/4/JARU |
| 247 | 9252 | ALTAR 84/AE.SQ//2*OPATA/3/PIFED |
| 248 | 9253 | KRICHAUFF/2*PASTOR |
| 249 | 9254 | KABY//2*ALUBUC/BAYA |
| 250 | 9255 | CNO79//PF70354/MUS/3/PASTOR/4/CROC_1/AE.SQUARROSA (224)//OPATA |
| 251 | 9256 | BUC/MN72253//PASTOR/3/BAV92 |
| 252 | 9257 | SCA/AE.SQUARROSA (409)//PASTOR/3/PASTOR |
| 253 | 9258 | CROC_1/AE.SQUARROSA (224)//OPATA/3/BJY/COC//PRL/BOW/4/BJY/COC//PRL/BOW |
| 254 | 9259 | CHEN/AE.SQ//2*OPATA/3/BAV92/4/JARU |
| 255 | 9260 | TIE CHUAN 1*2/3/HE1/3*CNO79//2*SERI |
| 256 | 9261 | ALTAR 84/AEGILOPS SQUARROSA (TAUS)//OPATA/3/ATTILA |
| 257 | 9262 | OASIS/5*BORL95/5/CNDO/R143//ENTE/MEXI75/3/AE.SQ/4/2*OCI |
| 258 | 9263 | PASTOR//TRAP#1/BOW/3/CHEN/AEGILOPS SQUARROSA (TAUS)//BCN |
| 259 | 9264 | PRL/2*PASTOR |
| 260 | 9265 | ND643/2*WAXWING |
| 261 | 9266 | ND643//2*PRL/2*PASTOR |
| 262 | 9267 | KIRITATI//2*PRL/2*PASTOR |
| 263 | 9268 | KIRITATI//2*ATTILA*2/PASTOR |
| 264 | 9269 | SAAR/WBLL1 |
| 265 | 9270 | CHONTE |
| 266 | 9271 | CHEWINK |
| 267 | 9272 | WHEAR/KIRITATI/3/C80.1/3*BATAVIA//2*WBLL1 |
| 268 | 9273 | WHEAR/4/SNI/TRAP#1/3/KAUZ*2/TRAP//KAUZ/5/C80.1/3*BATAVIA//2*WBLL1 |
| 269 | 9274 | WHEAR/KUKUNA/3/C80.1/3*BATAVIA//2*WBLL1 |
| 270 | 9275 | WHEAR/JARU/3/C80.1/3*BATAVIA//2*WBLL1 |
| 271 | 9276 | WHEAR/TUKURU/3/C80.1/3*BATAVIA//2*WBLL1 |
| 272 | 9277 | WHEAR/KURUKU/3/C80.1/3*BATAVIA//2*WBLL1 |
| 273 | 9278 | WHEAR//2*PRL/2*PASTOR |
| 274 | 9279 | WHEAR//2*PRL/2*PASTOR |
| 275 | 9280 | CNDO/R143//ENTE/MEXI_2/3/AEGILOPS SQUARROSA (TAUS)/4/WEAVER/5/2*KAUZ/6/PRL/2*PASTOR |
| 276 | 9281 | PRL/2*PASTOR/4/CHOIX/STAR/3/HE1/3*CNO79//2*SERI |
| 277 | 9282 | PRL/2*PASTOR/4/CHOIX/STAR/3/HE1/3*CNO79//2*SERI |
| 278 | 9283 | PFAU/MILAN/5/CHEN/AEGILOPS SQUARROSA (TAUS)//BCN/3/VEE#7/BOW/4/PASTOR |
| 279 | 9284 | PRL/SARA//TSI/VEE#5/3/TILHI/4/ATTILA/2*PASTOR |
| 280 | 9285 | ELVIRA/5/CNDO/R143//ENTE/MEXI75/3/AE.SQ/4/2*OCI/6/VEE/PJN//KAUZ/3/PASTOR |
| 281 | 9286 | HEILO//MILAN/MUNIA |
| 282 | 9287 | KAUZ//ALTAR 84/AOS/3/MILAN/KAUZ/4/HUITES |
| 283 | 9288 | QUAIU #1 |
| 284 | 9289 | PAURAQUE |
| 285 | 9290 | FRET2*2/BRAMBLING |
| 286 | 9291 | BECARD |
| 287 | 9292 | CROC_1/AE.SQUARROSA (205)//BORL95/3/PRL/SARA//TSI/VEE#5/4/FRET2 |
| 288 | 9293 | CROC_1/AE.SQUARROSA (205)//BORL95/3/PRL/SARA//TSI/VEE#5/4/FRET2 |
| 289 | 9294 | BETTY/3/CHEN/AE.SQ//2*OPATA |
