# Supplementary material for: Genetic characterization for lesion mimic and other traits in relation to spot blotch resistance in spring wheat
Source: PLoS One. 2020 Oct 5;15(10):e0240029. doi: 10.1371/journal.pone.0240029 (PMC7535040; doi:10.1371/journal.pone.0240029)
Supplement: Attachment — Submitted filename: Response to Reviewer 8th Oct 2019.doc [file pone.0240029.s004.doc]

ONE-D-19-19629
Genetic Characterization for Lesion mimic and other Traits in Relation to Spot blotch Resistance in Spring Wheat
PLOS ONE


Reviewers' comments:

Reviewer's Responses to Questions

**Comments to the Author**

1. Is the manuscript technically sound, and do the data support the conclusions?

The manuscript must describe a technically sound piece of scientific research with data that supports the conclusions. Experiments must have been conducted rigorously, with appropriate controls, replication, and sample sizes. The conclusions must be drawn appropriately based on the data presented.

Reviewer #1: Partly

Reviewer #2: Partly

2. Has the statistical analysis been performed appropriately and rigorously?

Reviewer #1: Yes

Reviewer #2: No

3. Have the authors made all data underlying the findings in their manuscript fully available?

Reviewer #1: Yes

Reviewer #2: No

4. Is the manuscript presented in an intelligible fashion and written in standard English?

PLOS ONE does not copyedit accepted manuscripts, so the language in submitted articles must be clear, correct, and unambiguous. Any typographical or grammatical errors should be corrected at revision, so please note any specific errors here.

Reviewer #1: No

Reviewer #2: No

5. Review Comments to the Author

Please use the space provided to explain your answers to the questions above. You may also include additional comments for the author, including concerns about dual publication, research ethics, or publication ethics. (Please upload your review as an attachment if it exceeds 20,000 characters)

Reviewer #1: Spot blotch is an important wheat disease in tropical and sub-tropical regions, especially India, China, Mexicao, etc. Identifying genetic loci responsible for Spot blotch resistance are important for wheat breeding and also for following genomic analysis. The manuscript performed genome wide association analysis of spot blotch (AUDPC) and lesion mimic (Lm) as well as leaf tip necrosis (Ltn), glaucousness index (GI) and latent period (LP) using a 289 GWAS panel and SNP assay. Positive association between AUDPC of spot blotch and Lm was found and negative correlation between Lm and GI, Ltn and LP were observed. SNP loci associated with Lm, AUDPC of spot blotch, were also identified. These information are valuable for wheat breeding and further fine gene mapping and cloning.

However, this manucript didn't conduct further analysis of their findings using currently available wheat genomic sequences resources which makes the current manusciprt less informative for the community. The authors should perform further analysis of the identified SNP loci, for example Lm on 6A (90-91 cM), AUDPC on 5A and 5B, etc.) in putative gene annotation and breeder friendly markers (like KASP) markers development and validation.

In addition, the authors found the AUDPC of spot blotch associated SNPs on 5A and 5B are derived from the same sets of probes. This should be explained further to clearify if these two locus on 5A and 5B are orthologous? or just missclassification of SNP signals of the same probe.

*Reply: As suggested we have tried to further analysis using currently available wheat genomic resources. We have identified candidate genes for significant marker trait association and carried out their detailed annotation and gene ontology.*

*In addition we also addressed your valuable concern (i.e. whether AUDPC of spot blotch associated SNPs on 5A and 5B are derived from the same sets of probes?). We have gone through it carefully; it was observed that there is no orthologous relation between mentioned locus on 5A and 5B. It may be just a miss classification of probe.*

Reviewer #2: The manuscript presents results aiming at analysing relationships between several wheat traits (lesion mimic symptoms, leaf tip necrosis symptom, glaucousness index, latent period, and resistance to spot blotch), and identifying markers associated to those traits. Several issues prevent publication at this stage, they are indicated below.

1) Materials and methods need to be described with more details. For example, the rationale of the choice of varieties used needs to be described.

*Reply: The studied population named WAMI germplasm, which is a set of 289 diverse wheat population, provided by Cimmyt, Mexico. Details of the lines are open access 37.*

2) Because the description of disease assessment is not sufficiently described, one cannot assess if the analyses conducted from these assessments are correct. More specifically, the "2-digit" scale needs to be explicitly described. It may be that this scale mixes two very different assessments, and that the analysis conducted is not correct. This potential issue needs to be thoroughly addressed.

*Reply: I have added* detailed *in the concerned para of material methods. It is based upon [34] i.e., Saari and Prescott 1975*

3) The Introduction, Results, and Discussion sections need to be improved, see detailed comments below.

*Reply: Thank you. All the correction incorporated.*
 The text needs to be edited. I provide examples for the beginning of the manuscript, starting at the Introduction, in order to illustrate the editing work required for the whole manuscript: L51 "Lesion mimic are necrotic symptoms…" : should be "Lesion mimic is …"; L52: HR needs to be spelled out the first time it is used; L53-4; there should be comas between te different items; L54: a space is missing before "Lm". Lm needs to be spelled out the first time it is used; L56: "restricts", not "restrict"; L57: "in the case" not "in case"

More detailed comments are given below.

L53-4: are these all examples with biotroph pathogens? This should be stated early.

*Reply:These all are examples of lesion mimic presence and expression in different plants without presence of any pathogen.*

L59: is it specifically about Lm plants?

*Reply: Yes, it is specifically about Lm plants which shown resistance against biotrophs but may facilitate hemibiotrophs and necrotrophs development.*

L60: what is ltn?

*Reply: Described in concern para. Ltn is denoted for Leaf tip necrosis which is an adult plant resistance character provides resistance against rust and spot blotch.*

L63: the rust(s) involved need to be indicated here

*Reply: Rust races like, leaf rust (Puccinia recondite sp. tritici and stripe rust (Puccinia striiformis)*

L65: Spell out the latin name in first instance.

*Reply: Added* as *Triticum aestivum* L.

L65-67: this is too vague and needs more details.

*Reply: Agreed. Explained in detail.*
L70: this needs rephrasing.

*Reply: Agreed. Rephrased*
L70-72: this needs rephrasing as the logic is not sound; do QTL refer to QTL for lm or for resistance to spot blotch?

*Reply: Here QTLs refers QTLs associated with resistance against Fusarium head blight and stem rust in wheat.*
L73: what is LP? If this is latency period, why choose this "trait" and not for example infection efficiency?

*Reply: Latency period was chosen after assumption of positive association of Lm and spot blotch. it was hypothesized that the varieties with longer LP and higher GI are resistance against spot blotch, can also restrict the HR Production of Lm as both HR may follow same pathway in host and can check with same mechanism which may be impart by genes responsible for longer LP and higher GI. It may be a selection criteria for varieties less or no Lm expression.*

4: Introduction: the introduction needs to be re-worked so that the reader can understand why this study was undertaken. For example, what is known or what can be hypothesized from the literature, about relationships between Lm, Ltn, and resistance to spot blotch. For a example, it is not indicated if spot blotch is considered as a necrotroph or biotroph, and this is needed. Actually, moving the first paragraph of Discussion in the Introduction, and reorganizing this section, could be a way to improve the Introduction.

*Reply: Agreed. Explained in introduction.*

L80-3: why were these varieties chosen, on what basis?

*Reply: WAMI population is formed by CIMMYT, Mexico consisted of elite genetically diverse germplasm.*
L101: the title needs to be changed

*Reply: Agreed. Title changed.*

L102-3: the two first sentences seem contradictory; was Lm assessed once or several times?

*Reply: Field was observed continuously after germination. Once Lm was expressed it was scored for three times at three different growth stages.*

L103: what was the sampling design for Lm symptoms assessment? How many flag leaves per elementary plot?

*Reply: Alpha lattice design, it was scored on flag leaf at the growth stage (GS) 69, 77 and 85*
L105-6: what is the unit: % of what?

*Reply: Here % area denoted the leaf area necrosis after HR of Ltn expression.*

L106: what was the sampling design?

*Reply: Alpha lattice design*
L108: what is the unit: % of what?

*Reply: Here % area denoted the leaf area necrosis after HR of Lm expression.*

L109: what is GI? This is not mentioned in the introduction. Why is it interesting to measure this trait?

*Reply: Explained in introduction. Glaucousness or waxiness on plants generally expressed at the time of flowering on the peduncle and flag leaf sheath.This trait restricts HR expansion of spot blotch, on that basis it was hypothesized that it can also check the expansion of HR of Lm and made it a trait of interest to study .*

L110: on what basis was this assessment made? Is there a diagram? Or is there an operational description for "most waxiness expression"?

*Reply: This sentence is creating confusion. We have just modified it.*

L12: the method used to measure LP is needs to be described, as well as the sampling design.

*Reply: Agreed, described in concern para as LP, the period between inoculations to spore production (in terms of days), and was recorded on five tagged plants in each replication.*

L116-8: the computation of "percent disease severity" needs to be clearly described here.

Reply: *have added* detailed *in the concerned para of material methods and hope now it would clarify the method of calculating AUDPC.*
Tables 1 and 2: what are the values given in the tables?

*Reply: detailed added in table.*
L179-80: Figure 2 should be described in more details.

*Reply: Described.*
Figure 3: more details are needed as footnotes of the figure; X and y axes need to be described.

*Reply: Detail is provided.*
Table 3: R2 values seem very small; can this translate into meaningful effects of phenotypes?
*Reply: R2 is low but it does not necessarily mean that will not translate into meaningful effects of phenotype. There are many studies reported earlier where R2 is low but still contributing to phenotypes. Details justification regarding this has been provided below.*

L263 This should appear in the Introduction as well

*Reply: Agreed, added in introduction.*
L265-6: this statement needs to be pondered with the other positive effects of Lm versus other wheat diseases.

*Reply*: *Most of the studies on Lm and pathogens are based on biotroph pathogens and reports are about its negative association with concern pathogen.*

L267: higher LP is not "providing protection", but is a consequence of other mechanisms which increase LP.

*Reply: here we stated that higher LP in association with GI and Ltn found protective against the disease and Lm expansion. We found that genotypes with higher LP in association with Ltn and good waxiness impart negative effect on Lm and spot blotch.*
L268-70: this is an hypothesis, which is not correct for LP: high LP is a consequence of other mechanisms, not a mechanism which will per se reduce the expression of Lm gene.

*Reply: Here, we concluded after our study on Lm and LP, our data showed that genotypes with higher LP restricted expansion of HR of Lm.*
L272: this is an hypothesis; correlation does not indicate any underlying mechanisms, but indicates only association.

*Reply: Any association or correlation (if seems important), which is consistence in expression in natural condition could be an important way or parameter to measure the associated characters or to sort out the associated problem so can be a selection criteria in plant breeding and genetics. The underlying mechanism with those associated characters needs to be work out. Here this study was based on three years of trial in two replication, we tried to come out with these conclusion based on our result we got.*

L284-307: a discussion on low R2 values is needed here.

*Reply: Although R^2^ is very low for the identified markers but p-value for all the markers are significant enough (p-value< 1e-03). As we have fitted mixed linear model between markers and phenotype and we know that in MLM R^2^ value does not play a major role. Also markers are correlated to each other causing multicollinearity leads to inflation in R^2^ value. This is a general trend in case of plant science where one can find such type of results. There are other studies reported earlier where marker R_2_ is very low but with low p-value hence contributing to phenotypes (Zegeye et al. 2014; Gyawali et al. 2017; Liu et al. 2017; Muleta et al. 2017; Ye et al. 2019)*

6. PLOS authors have the option to publish the peer review history of their article ([what does this mean?](https://journals.plos.org/plosone/s/editorial-and-peer-review-process#loc-peer-review-history)). If published, this will include your full peer review and any attached files.


If you choose “no”, your identity will remain anonymous but your review may still be made public.


**Do you want your identity to be public for this peer review?** For information about this choice, including consent withdrawal, please see our [Privacy Policy](https://www.plos.org/privacy-policy).

Reviewer #1: Yes: Zhiyong Liu

Reviewer #2: No


[NOTE: If reviewer comments were submitted as an attachment file, they will be attached to this email and accessible via the submission site. Please log into your account, locate the manuscript record, and check for the action link "View Attachments". If this link does not appear, there are no attachment files to be viewed.]


While revising your submission, please upload your figure files to the Preflight Analysis and Conversion Engine (PACE) digital diagnostic tool, <https://pacev2.apexcovantage.com/>. PACE helps ensure that figures meet PLOS requirements. To use PACE, you must first register as a user. Registration is free. Then, login and navigate to the UPLOAD tab, where you will find detailed instructions on how to use the tool. If you encounter any issues or have any questions when using PACE, please email us at [figures@plos.org](mailto:figures@plos.org). Please note that Supporting Information files do not need this step.

*In compliance with data protection regulations, you may request that we remove your personal registration details at any time.* [*(Remove my information/details)*](https://www.editorialmanager.com/pone/login.asp?a=r)*. Please contact the publication office*
