# Supplementary material for: Genetic characterization for lesion mimic and other traits in relation to spot blotch resistance in spring wheat
Source: PLoS One. 2020 Oct 5;15(10):e0240029. doi: 10.1371/journal.pone.0240029 (PMC7535040; doi:10.1371/journal.pone.0240029)
Supplement: Attachment — Submitted filename: Response to Reviewer Shweta et al 19 Dec 2019.doc [file pone.0240029.s005.doc]

ONE-D-19-19629
Genetic Characterization for Lesion mimic and other Traits in Relation to Spot blotch Resistance in Spring Wheat
PLOS ONE


Dear Dr. Manoj,

I am grateful you for giving me a chance to clarify the queries for the improving this paper as per standard of reputed journal ‘PLOS ONE’. I am also thankful to the reviewers who devoted much time and theirs inputs definitely improved the quality of paper. I hope you and reviewers will be satisfied with my inputs for each of your queries given here.

Reviewers' comments:

Reviewer's Responses to Questions

**Comments to the Author**

1. If the authors have adequately addressed your comments raised in a previous round of review and you feel that this manuscript is now acceptable for publication, you may indicate that here to bypass the “Comments to the Author”section, enter your conflict of interest statement in the “Confidential to Editor” section, and submit your "Accept" recommendation.

Reviewer #1: All comments have been addressed

Reviewer #2: (No Response)

2. Is the manuscript technically sounds, and do the data support the conclusions?

The manuscript must describe a technically sound piece of scientific research with data that supports the conclusions. Experiments must have been conducted rigorously, with appropriate controls, replication, and sample sizes. The conclusions must be drawn appropriately based on the data presented.

Reviewer #1: Yes

Reviewer #2: Yes

3. Has the statistical analysis been performed appropriately and rigorously?

Reviewer #1: Yes

Reviewer #2: Yes

4. Have the authors made all data underlying the findings in their manuscript fully available?

The PLOS Data policy requires authors to make all data underlying the findings described in their manuscript fully available without restriction, with rare exception (please refer to the Data Availability Statement in the manuscript PDF file). The data should be provided as part of the manuscript or its supporting information, or deposited to a public repository. For example, in addition to summary statistics, the data points behind means, medians and variance measures

should be available. If there are restrictions on publicly sharing data—e.g. participant privacy or use of data from a third party—those must be specified.

Reviewer #1: Yes

Reviewer #2: No

5. Is the manuscript presented in an intelligible fashion and written in standard English?

PLOS ONE does not copyedit accepted manuscripts, so the language in submitted articles must be clear, correct, and unambiguous. Any typographical or grammatical errors should be corrected at revision, so please note any specific errors here.

Reviewer #1: Yes

Reviewer #2: No

6. Review Comments to the Author

Please use the space provided to explain your answers to the questions above. You may also include additional comments for the author, including concerns about dual publication, research ethics, or publication ethics. (Please upload your review as an attachment if it exceeds 20,000 characters)

Reviewer #1: The authors revised the manuscript based on the reviewers' comments and suggestions. The quality of the manuscript was improved and I have no further comments.

Reviewer #2: I have acted as Reviewer #2 in the previous round of assessment of the manuscript. I have now focused on modifications made by the authors on the basis of the comments I have made. There are still a several issues to address, I indicate them below.

1) Rationale of the choice of varieties

Reviewer comment: this comment has not been addressed in the revised manuscript. Again, the comment was about the rationale of the choice of the varieties. This should be described.

*Reply: Added in manuscript in material and method section. We chose the (Wheat Association Mapping) WAM population (CIMMYT Collection) for our study because it is a set of 289 diverse germplasm containing considerable genotypic and phenotypic variations. Since these are germplasm stocks so characters chosen for study are stable on both genotypic and phenotypic level, so in general there is no question of segregation or elimination of traits under examine.*

2) Comments on further comments:

L60: Ltn was spelled out, but with about 5 edit mistakes: this should be: ". Another trait, leaf tip necrosis (Ltn), "

*Reply: Agreed. Suggestion incorporated.*

L63: the comment was addressed, but with mistakes: please edit and use the correct latin name for pathogen causing leaf rust and stripe rust.

*Reply: Agreed. Mistakes were edited and corrected.*

L65: I was referring to giving the full name instead of B. in B. sorokiniana; please provide the full name.

*Reply: Agreed, full name was added as Bipolaris* *sorokiniana instead of B. sorokiniana.*

L65-7: I did not see any change in the revised manuscript, in spite of the authors indicating otherwise in there answer to my comment.

*Reply: Agreed. Explained in detail.*

L70: The phrase has been changed, but now has additional edit mistakes (spaces missing).

*Reply: Agreed, correction incorporated.*

L70-2: although the sentence was rephrased, I still do not see the logics. Please rephrase. Do you mean that QTLs for resistance against head blight and stem rust were identified, and associated to Lm, while QTLs have not been identified in the case of resistance against spot blotch associated to Lm? What is also is not indicated, and what should be clearly stated, is if Lm has been associated to resistance against spot blotch on a phenotypic basis.

*Reply: Here the sentence stated that “QTLs for resistance against head blight and stem rust were identified, and were associated to Lm”, The QTLs studies were carried out for Lm against these diseases due to protective nature of Lm (has been associated to resistance against these disease on a phenotypic basis) means they were negatively associated with Lm. While The present study we are describing in text, shown the positive association of Lm with Spot blotch, also there is no such QTLs reported in the case of spot blotch associated to Lm. This study was carried out to study the effect of Lm on spot blotch.*

Title: "Scoring .." remove "disease" because this is addressed in the next sub-section

Assessments: additional information is needed:

*Reply: Agreed, correction incorporated according to suggestions.*

- For Ltn, on what leaves were the assessments made?

*Reply: It was scored on flag leaves. (Now mentioned in concern Para).*

- For glaucousness, a description of the 5 classes needs to be included.

*Reply: Agreed, scale described in related Para.*

- describe how LP was measured, from what observations; what was the frequency of observation? a reference is not sufficient.

*Reply: Description added in concern Para.*

L267: I insist, this statement is not correct. It should be changed as for example: "The results of this study showed that higher LP is associated to lower levels of spot blotch and Lm."

*Reply: Agreed, sentence modified according to suggestion.*

Looking at Table 2, the R2 values between GI and AUDPC

and Lm are very small and should not in my view be emphasised in the Discussion.

*Reply: R values are small but significant hence discussed.*

3) Editing the text

(1) Many occurrences where a space is needed; this needs to be corrected throughout the text.

*Reply: Correction made throughout the text and tries best to rectify it.*

(2) There are still many editorial mistakes which need to be addressed throughout the text.

*Reply: Agreed and mistakes were corrected in main text.*

7. PLOS authors have the option to publish the peer review history of their article (what does this mean?). If published, this will include your full peer review and any attached files.

If you choose “no”, your identity will remain anonymous but your review may still be made public.

**Do you want your identity to be public for this peer review?** For information about this choice, including consent

withdrawal, please see our Privacy Policy.

Reviewer #1: No

Reviewer #2: No

[NOTE: If reviewer comments were submitted as an attachment file, they will be attached to this email and accessible via

the submission site. Please log into your account, locate the manuscript record, and check for the action link "View

Attachments". If this link does not appear, there are no attachment files to be viewed.]

While revising your submission, please upload your figure files to the Preflight Analysis and Conversion Engine (PACE)

digital diagnostic tool, https://pacev2.apexcovantage.com/. PACE helps ensure that figures meet PLOS requirements. To

use PACE, you must first register as a user. Registration is free. Then, login and navigate to the UPLOAD tab, where you

will find detailed instructions on how to use the tool. If you encounter any issues or have any questions when using PACE,

please email us at figures@plos.org. Please note that Supporting Information files do not need this step.

*In compliance with data protection regulations, you may request that we remove your personal registration details at any time. (Remove my*

*information/details). Please contact the publication office if you have any questions.*
