# Supplementary material for: Genetic characterization for lesion mimic and other traits in relation to spot blotch resistance in spring wheat
Source: PLoS One. 2020 Oct 5;15(10):e0240029. doi: 10.1371/journal.pone.0240029 (PMC7535040; doi:10.1371/journal.pone.0240029)
Supplement: Attachment — Submitted filename: Response to reviewer Shweat et al. 2020.docx [file pone.0240029.s006.docx]

ONE-D-19-19629
Genetic Characterization for Lesion mimic and other Traits in Relation to Spot blotch Resistance in Spring Wheat
PLOS ONE


Dear Dr. Manoj,

I am grateful you for giving me a chance to clarify the queries for the improving this paper as per standard of reputed journal ‘PLOS ONE’. I am also thankful to the reviewers who devoted much time and theirs inputs definitely improved the quality of paper. I hope you and reviewers will be satisfied with my inputs for each of your queries given here.

Reviewers' comments:

Reviewer's Responses to Questions

**Comments to the Author**

1. If the authors have adequately addressed your comments raised in a previous round of review and you feel that this

manuscript is now acceptable for publication, you may indicate that here to bypass the “Comments to the Author”

section, enter your conflict of interest statement in the “Confidential to Editor” section, and submit your "Accept"

recommendation.

Reviewer #1: All comments have been addressed

Reviewer #2: (No Response)

2. Is the manuscript technically sound, and do the data support the conclusions?

The manuscript must describe a technically sound piece of scientific research with data that supports the conclusions. Experiments must have been conducted rigorously, with appropriate controls, replication, and sample sizes. The conclusions must be drawn appropriately based on the data presented.

Reviewer #1: Yes

Reviewer #2: Yes

3. Has the statistical analysis been performed appropriately and rigorously?

Reviewer #1: Yes

Reviewer #2: Yes

4. Have the authors made all data underlying the findings in their manuscript fully available?

The PLOS Data policy requires authors to make all data underlying the findings described in their manuscript fully available without restriction, with rare exception (please refer to the Data Availability Statement in the manuscript PDF file). The data should be provided as part of the manuscript or its supporting information, or deposited to a public repository. For example, in addition to summary statistics, the data points behind means, medians and variance measures should be available. If there are restrictions on publicly sharing data—e.g. participant privacy or use of data from a third party—those must be specified.

Reviewer #1: Yes

Reviewer #2: Yes

5. Is the manuscript presented in an intelligible fashion and written in standard English?

PLOS ONE does not copyedit accepted manuscripts, so the language in submitted articles must be clear, correct, and unambiguous. Any typographical or grammatical errors should be corrected at revision, so please note any specific errors here.

Reviewer #1: Yes

Reviewer #2: No

6. Review Comments to the Author

Please use the space provided to explain your answers to the questions above. You may also include additional comments for the author, including concerns about dual publication, research ethics, or publication ethics. (Please upload your review as an attachment if it exceeds 20,000 characters)

Reviewer #1: The authors addressed my concerns and I have no further comment! The manuscript was revised again based on another reviewers comment and the entire manuscript was improved.

Reviewer #2: I have acted as Reviewer #2 in the previous round of assessment of the manuscript. I have now focused on modifications made by the authors on the basis of the comments I have made. There are still a few issues to address, I indicate them below. The main issue I see, for which time will be required, is with respect to editing the English.

1) Rationale of the choice of varieties

This comment is now accounted for but further editing is needed. I suggest to replace the sentence "The population contains elite genetically diverse germplasm and so are stable for the traits under examine on both genotypic and phenotypic level and in general ruled out the chance of any segregation or elimination of charecters at maximum extent."

By the following sentence: "The population contains elite germplasm displaying considerable genetic and phenotypic variation. Furthermore, the population includes genotypes which are stable for the traits under examination."

*Reply: Agreed, Thanks for your kind suggestions, correction incorporated according to suggestions.*

2) Further comments:

The whole document should be revised thoroughly for editing, and to remove mistakes (e.g., space missing between two consecutive words).

*Reply: Agreed, it was due to different version of Microsoft word office.*

I am indicating additional specific comments are below:

L61: “Another trait, Leaf tip necrosis (Ltn),”: remove the symbols " at the beginning and at the end of this segment.

*Reply: Agreed, correction incorporated according to suggestions.*

L67: "resistance against spot blotch caused by Bipolaris sorokiniana ": add "the hemibiotroph pathogen" after "caused by"

*Reply: Agreed, correction incorporated according to suggestions.*

L68: add "increased" before "latent"

*Reply: Agreed, correction incorporated according to suggestions.*

L79-89: there are several problems here. One of them is that Lm is not associated to resistance vs fusarium head blight: the sentence needs to be changed and the reference 27 removed; FHB is caused by Fusarium spp. which are not biotrophic. Also, there are many sentences which repeat what was already written previously, and so which need to be removed. I propose to change this paragraph as follows: "Associations between Lm, Ltn, GI, and LP, and spot blotch have not been studied."

This sentence is to be followed by the beginning of the next paragraph.

*Reply: Agreed, reference no 27 removed and sentence reframed according to suggestions.*

L94: add " Ltn, GI, and LP" after "Lm"

*Reply: Agreed, correction incorporated according to suggestions.*

L94: replace "its" by "their"

*Reply: Agreed, word replaced according to suggestions.*

L123: remove "and disease"

*Reply: Agreed, word removed in main text.*

L130-131: delete "was recorded in the field"

*Reply: Agreed, was deleted in main text.*

L370: "LP also showed negative effect on Lm. Longer LP was not found favorable for the expression of Lm and contributed to enhance resistance against spot blotch": this needs to be changed: LP cannot be the cause for Lm. Please replace by "LP was negatively associated with Lm. Longer LP contributed to enhance resistance against spot blotch"

*Reply: Agreed, correction incorporated according to suggestions.*

L380-2: this needs rephrasing for English and for the meaning.

*Reply: Agreed, correction incorporated and paragraph rephrased according to suggestions.*

7. PLOS authors have the option to publish the peer review history of their article (what does this mean?). If published,

this will include your full peer review and any attached files.

If you choose “no”, your identity will remain anonymous but your review may still be made public.

**Do you want your identity to be public for this peer review?** For information about this choice, including consent

withdrawal, please see our Privacy Policy.

Reviewer #1: Yes: Zhiyong Liu

Reviewer #2: No

[NOTE: If reviewer comments were submitted as an attachment file, they will be attached to this email and accessible

via the submission site. Please log into your account, locate the manuscript record, and check for the action link "View

Attachments". If this link does not appear, there are no attachment files to be viewed.]

While revising your submission, please upload your figure files to the Preflight Analysis and Conversion Engine (PACE)

digital diagnostic tool, https://pacev2.apexcovantage.com/. PACE helps ensure that figures meet PLOS requirements.

To use PACE, you must first register as a user. Registration is free. Then, login and navigate to the UPLOAD tab, where

you will find detailed instructions on how to use the tool. If you encounter any issues or have any questions when

using PACE, please email us at figures@plos.org. Please note that Supporting Information files do not need this step.

*In compliance with data pr otection regulations, y ou may request*
