# Supplementary material for: Genetic characterization for lesion mimic and other traits in relation to spot blotch resistance in spring wheat
Source: PLoS One. 2020 Oct 5;15(10):e0240029. doi: 10.1371/journal.pone.0240029 (PMC7535040; doi:10.1371/journal.pone.0240029)
Supplement: Attachment — Submitted filename: Response to Reviewer 3 June 2020.docx [file pone.0240029.s007.docx]

PONE-D-19-19629R3

Genetic Characterization for Lesion mimic and other Traits in Relation to Spot blotch Resistance in Spring Wheat

PLOS ONE

Dear Dr. Manoj,

I am grateful you for giving me a chance to clarify the queries for the improving this paper as per standard of reputed journal ‘PLOS ONE’. I am also thankful to the reviewers who devoted much time and theirs inputs definitely improved the quality of paper. I hope you and reviewers will be satisfied with my inputs for each of your queries given here.

Reviewers' comments:

Reviewer's Responses to Questions

**Comments to the Author**

1. If the authors have adequately addressed your comments raised in a previous round of review and you feel that this manuscript is now acceptable for publication, you may indicate that here to bypass the “Comments to the Author” section, enter your conflict of interest statement in the “Confidential to Editor” section, and submit your "Accept" recommendation.

Reviewer #3: All comments have been addressed

Reviewer #4: (No Response)

2. Is the manuscript technically sound, and do the data support the conclusions?

The manuscript must describe a technically sound piece of scientific research with data that supports the conclusions. Experiments must have been conducted rigorously, with appropriate controls, replication, and sample sizes. The conclusions must be drawn appropriately based on the data presented.

Reviewer #3: Yes

Reviewer #4: Yes

3. Has the statistical analysis been performed appropriately and rigorously?

Reviewer #3: Yes

Reviewer #4: Yes

4. Have the authors made all data underlying the findings in their manuscript fully available?

The PLOS Data policy requires authors to make all data underlying the findings described in their manuscript fully available without restriction, with rare exception (please refer to the Data Availability Statement in the manuscript PDF file). The data should be provided as part of the manuscript or its supporting information, or deposited to a public repository. For example, in addition to summary statistics, the data points behind means, medians and variance measures should be available. If there are restrictions on publicly sharing data—e.g. participant privacy or use of data from a third party—those must be specified.

Reviewer #3: Yes

Reviewer #4: Yes

5. Is the manuscript presented in an intelligible fashion and written in standard English?

PLOS ONE does not copyedit accepted manuscripts, so the language in submitted articles must be clear, correct, and unambiguous. Any typographical or grammatical errors should be corrected at revision, so please note any specific errors here.

Reviewer #3: No

Reviewer #4: No

6. Review Comments to the Author

Please use the space provided to explain your answers to the questions above. You may also include additional comments for the author, including concerns about dual publication, research ethics, or publication ethics. (Please upload your review as an attachment if it exceeds 20,000 characters)

**Reviewer #3:** The study defines the genetic characterization of Lm, Ltn, GI, LP, and their association with spot blotch resistance in spring wheat. I could see that the manuscript was previously reviewed by two reviewers who had already commented twice and based on those comments, the manuscript was improved. I have gone through all the versions of the manuscript, reviewers’ comments and the response of authors. The work is scientifically robust and technically sound, as major flaws were addressed during the revisions. However, the authors were repeatedly requested to improve the language, which they were apparently not able to. There are several minor and major issues pertaining to sentence formation, choice of vocabulary, voice agreement and use/misuse of punctuation marks. Plos One does not copyedit accepted manuscripts, and therefore, the language in submitted articles must be clear, correct, and unambiguous. I strongly recommend the authors to take assistance from a professional editing service to resolve the language issues.

The certificate stating that the language was professionally edited should also be submitted to the journal for validation.

**Reviewer #4**: The manuscript is not well written. It needs language and scientific editing. I have highlighted some sentences in abstract that needs attention. Similar problems have been noticed i other portions of the manuscript. I think manuscript has good potential for publication in PLOS ONE but authors shall edit whole manuscript or get the manuscript edited from someone familiar with GWAS.

My feedback for Abstract:

1. The sentence < Lesion mimic (Lm) mutants are hypersensitive responses (HR) phenotype in the absence of the pathogen.> should be rewritten to make meaning clear

*Reply: Agreed, Changed the sentence accordingly.*

2. In sentence < In wheat, such mutants were reported to be resistant against rusts due to their biotrophic nature.> the authors shall mention clearly which rust because there are three common rust disease in wheat.

*Reply: Agreed, added in abstract.*

3. In sentence < In this study, 289 diverse wheat germplasm were phenotyped for 3 consecutive years (2012 to 2015).> the author shall write 289 genotypes or 289 wheat germplasm lines.

*Reply: Agreed, sentence was modified in main document, thanks for your kind suggestion.*

3. The sentence < Genotyping was done using Illumina iSelect beadchip assay for wheat having13589 highly polymorphic SNPs used for association mapping.> does not read well.

*Reply: Agreed, correction incorporated*

4. The authors wrote < In genome wise association study (GWAS),> but it is < In genome wide association study (GWAS),>

*Reply: Agreed, correction incorporated.*

6. < associated to various traits> may be changed with < associated with various traits>

similar problems have been noticed by me in all sections of the manuscript.

*Reply: Agreed, correction incorporated.*

General Comments:

1. The manuscript is not well written. It needs both language editing and scientific editing.

*Reply: A thorough English and science editing was done using co-authors and a professional Science Editor*

2. The results of GWAS are not intelligently presented. There is no mention about contribution of associated markers although same is presented in Table.

*Reply: Agreed, desired is incorporated.*

3. In structural analysis there is no mention about admixed genotypes although same could be seen in structural plot.

*Reply: Said information is incorporated in the manuscript.*

4. The quality of figures is poor

*Reply: Agreed, quality of figure is improved upto 300 dpi.*

5. Manhattan plot are poorly presented. The authors shall highlight the significant SNPs in Manhattan plots.

*Reply: Quality of Manhattan plot is improved, a threshold line at (-log_10_(P-value)=3) is drawn to highlight significant markers.*

6. The results of ANOVA are not properly discussed

*Reply: Agreed, results of ANOVA has been discussed in detail.*

7. PLOS authors have the option to publish the peer review history of their article (what does this mean?). If published,

this will include your full peer review and any attached files.

If you choose “no”, your identity will remain anonymous but your review may still be made public.

**Do you want your identity to be public for this peer review?** For information about this choice, including consent

withdrawal, please see our Privacy Policy.

Reviewer #3: No

Reviewer #4: No

[NOTE: If reviewer comments were submitted as an attachment file, they will be attached to this email and accessible via

the submission site. Please log into your account, locate the manuscript record, and check for the action link "View

Attachments". If this link does not appear, there are no attachment files to be viewed.]

While revising your submission, please upload your figure files to the Preflight Analysis and Conversion Engine (PACE)

digital diagnostic tool, https://pacev2.apexcovantage.com/. PACE helps ensure that figures meet PLOS requirements. To

use PACE, you must first register as a user. Registration is free. Then, login and navigate to the UPLOAD tab, where you

will find detailed instructions on how to use the tool. If you encounter any issues or have any questions when using PACE,

please email us at figures@plos.org. Please note that Supporting Information files do not need this step.
