# Supplementary material for: Genetic characterization for lesion mimic and other traits in relation to spot blotch resistance in spring wheat
Source: PLoS One. 2020 Oct 5;15(10):e0240029. doi: 10.1371/journal.pone.0240029 (PMC7535040; doi:10.1371/journal.pone.0240029)
Supplement: Attachment — Submitted filename: Response to Reviewers comments -21 Aug 2020.docx [file pone.0240029.s008.docx]

PONE-D-19-19629R3

Genetic Characterization for Lesion mimic and other Traits in Relation to Spot blotch Resistance in Spring Wheat

**Responses to Reviewers comments**

**Comments to the Author**

1. If the authors have adequately addressed your comments raised in a previous round of review and you feel that this manuscript is now acceptable for publication, you may indicate that here to bypass the “Comments to the Author” section, enter your conflict of interest statement in the “Confidential to Editor” section, and submit your "Accept"

recommendation.

Reviewer #3: All comments have been addressed

Reviewer #4: All comments have been addressed

2. Is the manuscript technically sound, and do the data support the conclusions?

The manuscript must describe a technically sound piece of scientific research with data that supports the conclusions. Experiments must have been conducted rigorously, with appropriate controls, replication, and sample sizes. The conclusions must be drawn appropriately based on the data presented.

Reviewer #3: Yes

Reviewer #4: Yes

3. Has the statistical analysis been performed appropriately and rigorously?

Reviewer #3: N/A

Reviewer #4: Yes

4. Have the authors made all data underlying the findings in their manuscript fully available?

The PLOS Data policy requires authors to make all data underlying the findings described in their manuscript fully available without restriction, with rare exception (please refer to the Data Availability Statement in the manuscript PDF file). The data should be provided as part of the manuscript or its supporting information, or deposited to a public repository. For example, in addition to summary statistics, the data points behind means, medians and variance measures should be available. If there are restrictions on publicly sharing data—e.g. participant privacy or use of data from a third

party—those must be specified.

Reviewer #3: Yes

Reviewer #4: Yes

5. Is the manuscript presented in an intelligible fashion and written in standard English?

PLOS ONE does not copyedit accepted manuscripts, so the language in submitted articles must be clear, correct, and unambiguous. Any typographical or grammatical errors should be corrected at revision, so please note any specific errors here.

Reviewer #3: Yes

Reviewer #4: Yes

6. Review Comments to the Author

Please use the space provided to explain your answers to the questions above. You may also include additional comments for the author, including concerns about dual publication, research ethics, or publication ethics. (Please upload your review as an attachment if it exceeds 20,000 characters)

Reviewer #3: Authors have addressed all the comments raised by the reviewers, and there is no further revisions required.

***Reply: Authors are the thankful for the positive feedback.***

Reviewer #4: The manuscript has been improved by keeping in view the comments made by reviewers but I think there are still language issues in the manuscript.

For instance In material and methods (Plant material section) LN 109-114 the authors are using past tense for as well future tense . The authors need to maintain consistency throughout manuscript.

***Reply: Agreed, the sentence improved accordingly and manuscript too. A thorough English and science editing was done using co-authors and a professional Science Edito. The certificate from professional writer is attached.***

In the same section the authors are still using <289 diverse wheat germplasm> despite suggestions were provided in previous revision.

***Reply: Agreed, the sentence improved accordingly and manuscript too.***

In addition I noticed that in table # 3 for LM, Gi etc the different associated markers are on same chromosomes with same position. Can authors have a look on the raw data again because having same position may signal same gene/QTL and not different genes/QTLs on that particular chromosome.

***Reply: Thanks for this good observation. However we have gone through raw data also and found that these are different SNPs (mapped at same genetic distance i.e. cM) and we***

***have also confirmed these results from earlier studies where multiple SNPs are located at same genetic distance (this may be due to reason that cM distance represents large chunks of genomic regions, sometimes in hundreds of KB).***

***However we have highlighted those SNPs which represent the same candidate genes in the annotation section of manuscript.***

7. PLOS authors have the option to publish the peer review history of their article (what does this mean?). If published, this will include your full peer review and any attached files.

If you choose “no”, your identity will remain anonymous but your review may still be made public.

**Do you want your identity to be public for this peer review?**

**Reply: *No.***
